# An observational study of the epidermal growth factor receptor-tyrosine kinase inhibitor resistance mechanism in epidermal growth factor receptor gene mutation-positive non-small cell lung cancer

**DOI:** 10.1097/MD.0000000000012660

**Published:** 2018-10-05

**Authors:** Akihiro Yoshimura, Junji Uchino, Keiko Tanimura, Yusuke Chihara, Nobuyo Tamiya, Yoshiko Kaneko, Takayuki Takeda, Osamu Hiranuma, Isao Hasegawa, Yutaka Kubota, Shinsuke Shiotsu, Chieko Takumi, Noriya Hiraoka, Tadaaki Yamada, Koichi Takayama

**Affiliations:** a Department of Pulmonary Medicine, Kyoto Prefectural University of Medicine; b Department of Respiratory Medicine, Uji-Tokushukai Medical Center; c Department of Respiratory Medicine, Otsu City Hospital; d Department of Respiratory Medicine, Japanese Red Cross Kyoto Daini Hospital; e Department of Respiratory Medicine, Japanese Red Cross Kyoto Daiichi Hospital, Japan.

**Keywords:** afatinib, EGFR mutation, EGFR-TKI resistance, NSCLC

## Abstract

Non-small cell lung cancer (NSCLC) patients with epidermal growth factor receptor (EGFR) mutation show a high response to EGFR-tyrosine kinase inhibitor (EGFR-TKI). Clinically, EGFR-positive NSCLC acquires several resistance mechanisms during EGFR-TKI treatment, such as the emergence of a secondary mutation (T790M), MET gene amplification, and transformation to small cell lung cancer. However, the mechanism of resistance to afatinib, a second-generation EGFR-TKI, remains unclear. In this study, we prospectively investigate the mechanism of resistance to afatinib using proteomic analyses.

In total, 35 EGFR-positive NSCLC patients of both sexes and ≥20 years old will be included. NSCLC patients with major obstacles in major organs, such as bone marrow, heart, lung, liver, and kidney, will be excluded. Eligible patients will be administered afatinib or gefitinib until disease progression and proteomic analysis will be performed with biopsy samples before treatment and at disease progression.

The primary outcome is to detect the potential predictive anomalies in proteins that can be candidates for the resistance factor of afatinib. The secondary outcome is to detect gene and protein abnormalities affecting progression-free survival, response rate, and rate of disease control in afatinib therapy.

The protocol was approved by the institutional review boards of Kyoto Prefectural University of Medicine and all the participating hospitals. Written informed consent was obtained from all patients before registration, in accordance with the Declaration of Helsinki. The results of the study will be disseminated via publications in peer-reviewed journals.

Trial registration number is UMIN000031013.

## 1 Introduction

In recent years, treatment advances against non-small cell lung cancer (NSCLC) with driver mutations in genes, such as epidermal growth factor receptor (EGFR), have revealed that patients with driver mutations show greater responses to tyrosine kinase inhibitors (TKIs) compared to chemotherapy.^[1]^ Among EGFR-positive NSCLC patients, treatment with EGFR-TKIs showed significantly longer progression-free survival (PFS) compared to platinum doublet therapy.^[2–5]^

Afatinib, a second-generation EGFR-TKI, is an irreversible ErbB family binder and blocker that inhibits EGFR, HER2, and HER4.^[6–8]^ At present, gefitinib, erlotinib, and afatinib have been approved for EGFR-positive NSCLC patients as a first-line treatment. For the first time, afatinib among EGFR-TKIs showed significantly longer PFS and overall survival compared to platinum doublet therapy in patients with deletion of exon 19 in EGFR.^[9]^ Moreover, afatinib significantly prolonged the PFS compared to gefitinib.^[10]^ On the other hand, strong digestive organ toxicity and dermal toxicity were observed; therefore, afatinib was not recommended in poor performance status and the elderly. However, by adjusting the amount of medicine to reduce these side effects, patients were reported to be able to continue afatinib therapy with a therapeutic effect equivalent to that reported previously in a study including the elderly.^[11]^

Almost all EGFR-positive NSCLC patients acquire several resistance mechanisms during EGFR-TKIs treatment, such as the emergence of a secondary mutation (T790M), MET gene amplification, and transformation to small cell lung cancer.^[12,13]^ Of these, EGFR-T790M is the most common resistance mechanism and found in approximately 50% of cases of resistance during the administration of first-generation EGFR-TKIs.^[12,14]^ Osimertinib, a third-generation EGFR-TKI, is approved for EGFR-T790M-positive NSCLC patients who experienced disease progression during the first and/or second EGFR-TKI treatment because the AURA-3 study reported that osimertinib significantly prolonged PFS compared to platinum-pemetrexed therapy in EGFR-T790M-positive NSCLC patients after resistance.^[15]^

Little is known regarding the presence of associations between the different responses to EGFR-TKIs and the emergence of T790M. As EGFR-T790M detection was reported to be associated with the initial EGFR-TKI duration and response rate,^[16]^ afatinib treatment is expected to be associated with the greater emergence of EGFR-T790M in basic and clinical research.

Recently, advances in the proteomic analysis have revealed the anomalies in genes and proteins in increased detail. At this time, we are prospectively investigating the tumor proteome before afatinib therapy and after disease progression using proteomic analysis. The primary endpoint is to explore anomalies in proteins that can be candidates for the resistance factor of afatinib. The secondary endpoint is to explore the protein abnormalities affecting the PFS, objective response rate (ORR), and disease control rate (DCR) in afatinib therapy.

## 2 Methods and analysis

### 2.1 Study design

**Figure 1:**
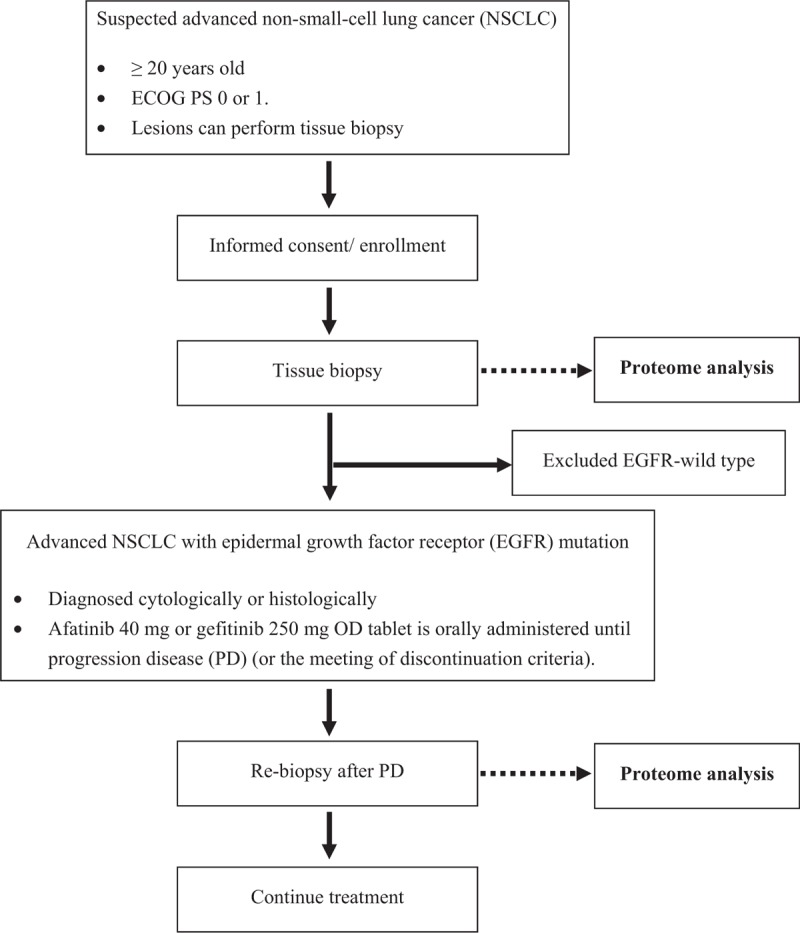
Study flowchart.

The study is a prospective observational study. Figure 1 depicts a flowchart of the study.

### 2.2 Study setting

Four hospitals agreed to participate in this study. The study protocol was approved by the institutional review board of each hospital. Written informed consent was obtained from all the patients before registration in accordance with the Declaration of Helsinki. The samples are being analyzed by the National Cancer Center Hospital. At least annual independent monitoring is planned in accordance with the Japanese clinical trial guidelines.

### 2.3 Participants

The inclusion criteria are as follows:

(1) Patients with histologically or cytologically confirmed stage IIIB/IV NSCLC and postoperative recurrence.
(2) Chemotherapy-naive patients planning for afatinib or first-generation EGFR-TKI treatment.
(3) Patients whose tissues can be harvested for the study before afatinib or first-generation EGFR-TKI treatment and resistance.
(4) Performance status (ECOG) 0 to 1.
(5) Patients with a life expectancy of at least 3 months.
(6) Patients whose consent has been obtained in the document for this study.
(7) Patients who are ≥20 years of age (at the time of enrollment).
(8) Patients for whom bone marrow, hepatic, and renal functions have all been confirmed as normal within 14 days before enrollment according to the following clinical test standards:
  1) WBC ≥ 3000/mm^3^ to ≤12,000/mm^3^,
  2) Neutrophil count ≥ 1500/mm^3^,
  3) Platelet count ≥ 100,000/mm^3^,
  4) Hemoglobin ≥ 9.0 g/dL,
  5) AST, ALT ≤ 100 IU/L,
  6) Total bilirubin ≤ 1.5 mg/dL,
  7) Creatinine ≤ 2.0 mg/dL,
  8) SpO_2_ (room air) ≥ 90%, and
  9) Participation in “prospective observational research (LC-SCRUM)” to clarify the clinicopathological and molecular biological characteristics of low-frequency genetic change positive lung cancer, such as presence of the RET fusion gene.

The exclusion criteria are as follows:

(1) Patients with pulmonary disorders, such as idiopathic pulmonary fibrosis, interstitial pneumonia, pneumoconiosis, active radiation pneumonitis, and drug-induced pneumonia,
(2) Patients with infectious disorders requiring intravenous injection of antibacterial drugs or antimycotics,
(3) Patients unable to swallow oral medications,
(4) Patients who are pregnant, nursing, or possibly pregnant,
(5) Patients with symptomatic brain metastases,
(6) Patients with active double cancer,
(7) Patients with uncontrollable diabetes mellitus,
(8) Patients with complications of clinical concern (such as uncontrollable cardiac disease, severe cardiac arrhythmia requiring medical treatment, and sustained serious diarrhea), and
(9) Any other patients who are regarded as unsuitable for this study by the investigators.

### 2.4 Dose and treatment regimens

Afatinib (40 mg) or gefitinib (250 mg) OD tablet is administered orally. Oral administration of these drugs is continued until disease progression or the criteria for discontinuation.

### 2.5 Rationale for the setting of the number of enrolled subjects

As this study is a prospective observational study for biomarker detection, the number of enrolled subjects is not set. Enrollment of 35 subjects who met the eligibility criteria until March 2020 was planned.

### 2.6 Proteomic analysis

Samples at diagnosis and upon rebiopsy after resistance are cryopreserved at −80°C after washing with physiological saline. If rebiopsy samples are diagnosed with NSCLC, proteomic analysis will be performed at National Cancer Center Hospital as follows: comprehensive protein expression analysis using mass spectrometry and exhaustive phosphoenzyme activity analysis using PamStation.

### 2.7 Statistical methods

PFS: survival curve, median (Kaplan–Meier method), confidence interval of the median (Brookmeyer and Crowley method), and standard error of the annual rate (Greenwood method).

ORR: response rate and its two-sided 95% confidence interval (Wilson method). Statistical significance is considered when the lower limit of the estimated confidence interval exceeds a threshold of 35%.

DCR: disease control rate and its two-sided 95% confidence interval (Wilson method).

### 2.8 Ethics

The trial received ethical approval from the Ethics Committee of Kyoto Prefectural University of Medicine, Kyoto, Japan (number: ERB-C-1106). The trial is subject to the supervision and management of the Ethics Committee.

### 2.9 Trial status

This study opened to recruitment in May 2018, with a planned last follow-up in March 2020. As of August 2018, 4 subjects have been enrolled.

## 3 Discussion

Ultimately, EGFR-positive NSCLC patients acquire resistance to EGFR-TKI during therapy. EGFR-T790M, which is found in approximately 50% of patients after resistance, is one of the several resistance mechanisms involved.^[12,14]^ If we detect T790M in rebiopsy samples, EGFR-T790M-positive patients could be administrated osimertinib. Several investigations have shown T790M was significantly detected in EGFR-positive NSCLC patients with a longer duration of initial EGFR-TKI and a higher response rate to initial EGFR-TKI. Afatinib differs from first-generation EGFR-TKIs in binding and blocking the ErbB family that inhibits EGFR, HER2, and HER4 irreversibly as shown in basic studies.^[6–8]^ Moreover, afatinib therapy was superior to gefitinib in clinical research with respect to PFS and ORR.^[10]^ The emergence of T790M might show a greater increase during afatinib therapy than during gefitinib therapy, while resistance mechanisms differing from T790M, MET amplification, and transformation to small cell lung cancer might emerge. Therefore, evaluation of the tumor proteome before treatment and after the acquisition of resistance using proteomic analysis is needed.

## Acknowledgments

This study is funded by Nippon Boehringer Ingelheim, Co., Ltd.

## Author contributions

**Conceptualization:** Akihiro Yoshimura, Junji Uchino, Keiko Tanimura, Tadaaki Yamada, and Koichi Takayama

**Data curation:** Akihiro Yoshimura

**Formal analysis:** Akihiro Yoshimura

**Funding acquisition:** Junji Uchino

**Investigation:** Akihiro Yoshimura, Junji Uchino, Keiko Tanimura, Yusuke Chihara, Nobuyo Tamiya, Yoshiko Kaneko, Takayuki Takeda, Osamu Hiranuma, Isao Hasegawa, Yutaka Kubota, Shinsuke Shiotsu, Chieko Takumi, Noriya Hiraoka, and Tadaaki Yamada

**Methodology:** Tadaaki Yamada

**Project administration:** Tadaaki Yamada and Koichi Takayama

**Supervision:** Koichi Takayama

**Writing – original draft:** Akihiro Yoshimura

Junji Uchino orcid: 0000-0003-0651-7767
